# Diversity of kidney care referral pathways in national child health systems of 48 European countries

**DOI:** 10.3389/fped.2024.1327422

**Published:** 2024-01-16

**Authors:** Velibor Tasic, Vidar O. Edvardsson, Evgenia Preka, Larisa Prikhodina, Constantinos J. Stefanidis, Rezan Topaloglu, Diamant Shtiza, Ashot Sarkissian, Thomas Mueller-Sacherer, Rena Fataliyeva, Ina Kazyra, Elena Levtchenko, Danka Pokrajac, Dimitar Roussinov, Danko Milošević, Avraam Elia, Tomas Seeman, Mia Faerch, Inga Vainumae, Janne Kataja, Michel Tsimaratos, Irakli Rtskhiladze, Peter F. Hoyer, George Reusz, Atif Awan, Danny Lotan, Licia Peruzzi, Nazim Nigmatullina, Nasira Beishebaeva, Edite Jeruma, Augustina Jankauskiene, Olivier Niel, Valerie Said-Conti, Angela Ciuntu, Snežana Pavićević, Michiel Oosterveld, Anna Bjerre, Marcin Tkaczyk, Ana Teixeira, Adrian C. Lungu, Alexey Tsygin, Vesna Stojanović, Ludmila Podracka, Tanja Kersnik Levart, Mar Espino-Hernández, Per Brandström, Giuseppina Sparta, Harika Alpay, Dmytro Ivanov, Jan Dudley, Komiljon Khamzaev, Dieter Haffner, Jochen Ehrich

**Affiliations:** ^1^ Medical School, University Children’s Hospital, Skopje, North Macedonia; ^2^ Iceland Children’s Medical Center, Landspitali – The National University Hospital of Iceland, Reykjavik, Iceland; ^3^ Paediatric Nephrology, University Hospital Southampton NHS Foundation Trust, Southampton, United Kingdom; ^4^ Research and Clinical Institute for Pediatrics, Pirogov Russian National Research Medical University, Moscow, Russia; ^5^ Department of Pediatric Nephrology, “Mitera” Children’s Hospital, Athens, Greece; ^6^ Department of Pediatric Nephrology, Hacettepe University School of Medicine, Ankara, Turkey; ^7^ Department of Pediatric Nephrology, University Hospital Centre “Mother Teresa”, Tirana, Albania; ^8^ Arabkir Joint Medical Centre, Yerevan State Medical University, Yerevan, Armenia; ^9^ Department of Pediatric Nephrology and Gastroenterology, Medical University of Vienna, Vienna, Austria; ^10^ Department of Pediatric Nephrology, Children’s Hospital, Baku, Azerbaijan; ^11^ 1st Department of Pediatrics, Belarusian State Medical University, Minsk, Belarus; ^12^ Department of Pediatrics & Division of Pediatric Nephrology, University Hospitals Leuven, Leuven, Belgium; ^13^ Department of Pediatric Nephrology, University Children’s Hospital, Sarajevo, Bosnia and Herzegovina; ^14^ Nephrology and Hemodialysis Clinic, University of Sofia, Sofia, Bulgaria; ^15^ Pediatric Clinic, University Hospital Center Zagreb, Zagreb, Croatia; ^16^ Department of Paediatrics, Archbishop Makarios III Hospital, Nicosia, Cyprus; ^17^ Department of Pediatrics, 2nd Medical Faculty, Charles University Prague, Prague, Czech Republic; ^18^ Department of Pediatrics and Adolescent Medicine, Aarhus University Hospital, Aarhus, Denmark; ^19^ Department of Pediatrics, University of Tartu, Tartu, Estonia; ^20^ Department of Paediatrics and Adolescents Medicine, Turku University Hospital, Turku, Finland; ^21^ Department of Multidisciplinary Pediatrics, Pediatric Nephrology Unit, Assistance Publique des Hôpitaux de Marseille, Marseille, France; ^22^ Department of Pediatrics, Medical Centre Mrcheveli, Tbilisi, Georgia; ^23^ Department of Pediatric Nephrology, University Hospital Essen, Essen, Germany; ^24^ First Department of Pediatrics, Semmelweis University, Budapest, Hungary; ^25^ Department for Paediatric Nephrology & Transplantation, Children's Health Ireland, Dublin, Ireland; ^26^ Division of Pediatric Nephrology, Sheba Medical Center, Edmond and Lily Children’s Hospital, Tel Hashomer, Israel; ^27^ Nephrology, Dialysis and Transplantation Unit, Regina Margherita University Children's Hospital, Turin, Italy; ^28^ Department of Nephrology, Kazakh National Medical University, Almaty, Kazakhstan; ^29^ Department of Nephrology, National Center of Maternity and Childhood Welfare under the Ministry of Health of the Kyrgyz Republic, Bishkek, Kyrgyzstan; ^30^ Bērnu Slimību Klīnika, Nefroloģijas Profila Virsārste, Riga, Latvia; ^31^ Pediatric Center, Institute of Clinical Medicine, Vilnius University, Vilnius, Lithuania; ^32^ Pediatric Nephrology Unit, Department of Pediatrics, Centre Hospitalier de Luxembourg, Luxembourg, Luxemburg; ^33^ Department of Child and Adolescent Health, Mater Dei Hospital, Msida, Malta; ^34^ Nephrology Unit, National Institute of Health Care for Mother and Child, Chisinau, Moldova; ^35^ Clinical Center of Montenegro, Institute for Sick Children, Podgorica, Montenegro; ^36^ Department of Paediatric Nephrology, Emma Children’s Hospital, Amsterdam University Medical Center, Amsterdam, Netherlands; ^37^ Department of Pediatric and Adolescent Medicine, University Hospital of Oslo, Oslo, Norway; ^38^ Department of Pediatrics, Immunology and Nephrology, Polish Mother’s Memorial Hospital Research Institute, Lodz, Poland; ^39^ Pediatric Nephrology Division, Centro Hospitalar Universitário do Porto, Porto, Portugal; ^40^ Pediatric Nephrology, Fundeni Clincal Institute, Bucharest, Romania; ^41^ National Medical and Research Centre for Children's Health, Moscow, Russia; ^42^ Pediatric Intensive Care Unit, Institute for Child and Youth Health Care of Vojvodina, Novi Sad, Serbia; ^43^ Department of Pediatrics, Comenius University, Bratislava, Slovakia; ^44^ Pediatric Nephrology Department, Children’s Hospital, University Medical Centre Ljubljana, Ljubljana, Slovenia; ^45^ Pediatric Nephrology, Pediatrics, University Hospital 12 Octubre, Madrid, Spain; ^46^ Pediatric Uro-Nephrologic Center, Department of Pediatrics Queen Silvia Children’s Hospital, Sahlgrenska University Hospital, Gothenburg, Sweden; ^47^ Department of Pediatric Nephrology, University Children’s Hospital, Zurich, Switzerland; ^48^ Division of Pediatric Nephrology, Marmara University, Istanbul, Turkey; ^49^ Nephrology and RRT Department, Shupyk National Healthcare University of Ukraine, Kyiv, Ukraine; ^50^ Department of Paediatric Nephrology, Bristol Children's Hospital, Bristol, United Kingdom; ^51^ Department of Pediatric Nephrology and Hemodialysis, Tashkent Pediatric Medical Institute, National Children's Medical Center, Tashkent, Uzbekistan; ^52^ Children’s Hospital, Hannover Medical School, Hannover, Germany

**Keywords:** pediatric nephrology, healthcare services, referral clinical pathways, urinary tract infections, nephrotic syndrome, acute kidney injury

## Abstract

**Background:**

Primary, secondary and tertiary healthcare services in Europe create complex networks covering pediatric subspecialties, sociology, economics and politics. Two surveys of the European Society for Paediatric Nephrology (ESPN) in 1998 and 2017 revealed substantial disparities of kidney care among European countries. The purpose of the third ESPN survey is to further identify national differences in the conceptualization and organization of European pediatric kidney health care pathways during and outside normal working hours.

**Methods:**

In 2020, a questionnaire was sent to one leading pediatric nephrologist from 48 of 53 European countries as defined by the World Health Organization. In order to exemplify care pathways in pediatric primary care nephrology, urinary tract infection (UTI) was chosen. Steroid sensitive nephrotic syndrome (SSNS) was chosen for pediatric rare disease nephrology and acute kidney injury (AKI) was analyzed for pediatric emergency nephrology.

**Results:**

The care pathways for European children and young people with urinary tract infections were variable and differed during standard working hours and also during night-time and weekends. During daytime, UTI care pathways included six different types of care givers. There was a shift from primary care services outside standard working hours to general outpatient polyclinic and hospital services. Children with SNSS were followed up by pediatric nephrologists in hospitals in 69% of countries. Patients presenting with community acquired AKI were admitted during regular working hours to secondary or tertiary care hospitals. During nights and weekends, an immediate shift to University Children's Hospitals was observed where treatment was started by intensive care pediatricians and pediatric nephrologists.

**Conclusion:**

Gaps and fragmentation of pediatric health services may lead to the risk of delayed or inadequate referral of European children with kidney disease to pediatric nephrologists. The diversity of patient pathways outside of normal working hours was identified as one of the major weaknesses in the service chain.

## 1 Introduction

While some pediatric kidney and urinary tract disorders can be treated in primary care, there will inevitably be children who require more specialized treatment options including hospitalization. The number of children with kidney disorders treated at secondary and tertiary care facilities depends on disease severity and the availability of advanced pediatric nephrology care. Two previous surveys conducted by members of the European Society for Paediatric Nephrology (ESPN) in the years 1998 and 2017 revealed a significant diversity in the availability and delivery of kidney care at the primary and tertiary care levels in the participating European countries (1, 2). In cooperation with the European Paediatric Association (EPA/UNEPSA) we described three different systems on how general primary healthcare for children under the age of 15 years is organized in European countries (3). First there is the primary pediatric care system (PPC) where more than 75% of the childhood population are under the care of pediatricians; then the general practitioner/family doctor system (GPC) where more than 75% of the children are first seen by general practitioners and thirdly, a mixed care system (MiC), in which a similar proportion of the child population is treated by either pediatricians or general practitioners. One of the main objectives of national healthcare policies are to reduce the rate of hospitalizations as they impose extra burden on both the children and their families and increase healthcare cost, particularly when treating less severe acute conditions (4). Significant delays in the hospitalization of children with severe kidney disease such as acute kidney injury can lead to potentially preventable complications and even death. As pediatric nephrologists are rarely involved in primary care, the above-described pre-defined clinical pathways (CPW) may delay timely patient referrals and thereby cause harm. Identifying the organizational gaps between the primary, secondary, and tertiary levels of care for children with kidney disease may facilitate timely referrals and prompt institution of appropriate treatment (4).

The objectives of the current study were to assess the organization of primary healthcare services, outpatient care following hospitalization, and referral pathways and the levels at which healthcare is provided for European children with kidney disease.

## 2 Materials and methods

### 2.1 Study design

This is a cross-sectional survey designed to assess organization of primary healthcare services, outpatient care following hospitalization, referral pathways and the level at which healthcare is provided for European children with kidney disease during regular working hours and during nights and weekends: (a) suspected urinary tract infections (UTI) were used for the study of available first contact services for children with kidney disease in the participating countries and how they matched with the previously reported pattern of European primary pediatric healthcare systems (4); (b) a first episode of steroid sensitive nephrotic syndrome (SSNS) was used for the study of outpatient care organization following hospitalization for children with kidney disease; and (c) suspected community acquired acute kidney injury (AKI) was used for the study of referral pathways for children with serious life-threatening kidney disorders from primary care to highly specialized referral institutions. Urinary tract infections, SSNS, and AKI are well defined pediatric kidney and urinary tract problems that most commonly are first seen by primary care physicians. These conditions are, therefore, ideal for the study of differences in the organization of pediatric kidney care pathways and national healthcare services between the different European countries.

### 2.2 Questionnaire

In 2020, a 15-question survey was designed by six ESPN members (V.T., L.P., E.P., C.J.S., R.T., and J.E.) to assess the organization of renal care in children. This article focuses on the responses to seven questions. All participants were asked to answer multiple-choice and open-ended questions. The questions about ESPN policy addressed workforce planning, health care delivery systems, the organization of outpatient and inpatient care for children with kidney disease, and changes needed to improve current treatment strategies for AKI. The questionnaire was accompanied by a letter explaining both the purpose of the project and the definitions of the three different primary health care systems for children. A single leading pediatric nephrologist from 48 of the 53 European countries (as defined by the World Health Organization in 2020) was selected by the authors and asked to represent his or her country and complete the questionnaire after consulting with colleagues as necessary. All 48 participants were members of ESPN, either presidents of national pediatric nephrology societies or senior pediatric nephrologists in highly specialized pediatric renal centers.

### 2.3 Participating countries

Representatives from 48 countries from Iceland in the west to Kazakhstan in the east and from Norway in the north to Malta in the south participated in the survey. Five European countries with a total population of less than 200,000 inhabitants were excluded from the study. In the selection of European countries for our study, we adhered to the definition of Europe in the list of the World Health Organization (WHO). The WHO Regional Office for Europe (WHO/Europe) is one of WHO's six regional offices around the world (5). It serves the WHO European Region which comprises 53 countries.

### 2.4 Data collection and storage

The survey was administered by e-mail communication and all the 48 invited experts agreed to their participation in the study. All respondents were fluent in the English language. Data were entered into the study database designed in Excel. Data completeness and accuracy assessment was conducted by VT and JE at the coordinating sites in Skopje and Hanover. In the case of incomplete data the respective survey participants were contacted and missing information collected. A brief outline of the survey is presented below while the complete questionnaire is detailed in the Addendum (Supplementary Material):

1. How is the first healthcare contact during and outside of regular working hours organised for children less than 18 years of age suspected of UTI?
2. How is follow-up healthcare for hospitalized children with SSNS organized?
3. How is emergency care organized for children suspected of AKI in the primary care setting?
4. What are the top three priorities for urgent change to improve current treatment strategies?

### 2.5 Statistical considerations

Data collected by the questionnaire were analyzed at the two coordinating sites. Data were analyzed using descriptive statistics and the chi-square test. For the purpose of analysis, countries were divided into groups based on (a) population size, (b) gross domestic product (GDP)/gross national product (GNP) per capita, (c) type of primary pediatric healthcare service systems (PPC, GPC, MiC), (d) political systems and (e) geographic region.

## 3 Results

A total of 48 (100%) nephrologists from the same number of European countries with more than 200,000 inhabitants completed the questionnaire. Completeness of information allowed comparing the predefined variables such as size of population, geographical regions and types of primary pediatric healthcare system or political system. The 48 represented countries belonged to Central (*n* = 7), Northern (*n* = 5), Southern (*n* = 11), Eastern (6) and Western Europe. (*n* = 6). Population size was <2 million in 6 countries, 2–3.6 million in 7 countries, 4–8 in 13 countries, 9–21 in 12 countries and >21 million people in 10 countries; Political systems included European Union (*n* = 24), former Soviet Union countries, (*n* = 13) and other political systems (*n* = 11). Of all 48 countries 20 were low-income, 12 were middle-income, and high-income, 4 no data.

### 3.1 Pediatric primary care nephrology: pathways of care for children suspected of urinary tract infections

During daytime hours, nighttimes and weekends ten different services were reported from 48 countries: (1) General practitioner, (2) primary care pediatrician, (3) nurse practitioner, (4) polyclinic (stand-alone), (5) emergency room in general or children's hospital, (6) emergency hospital, (7) emergency doctor with own car, (8) mobile ambulance car (state owned), (9) pediatric nephrologist, (10) telephone service.

During daytime hours, six different care pathways were observed for children suspected of UTI's which included primary care pediatricians (PCPeds), general practitioners (GPs), nurse practitioners, stand alone polyclinics, hospital outpatient services and emergency hospital. In 31% of countries, care for children suspected of UTIs was provided by primary care pediatricians and in 17% by general practitioners or a combination of both (17%). In 12 out of 13 countries with a primary child healthcare system supplied with PCPeds, the patients with UTI were primarily seen by pediatricians during daytime. During nighttimes and weekends 69% of countries offered one or more other services (Table 1). In 13 out of 14 European countries with a healthcare system supplied with GPs, the children with UTI were primarily seen by GPs during daytime. During nighttime and on weekends 57% of countries offered other services (Table 1). In 13 European countries with a mixed healthcare system, both PCPeds and GPs were involved in the care of children suspected of UTI during daytime. During nighttimes and weekends 69% of countries offered other services (Table 1). Daytime UTI care followed the distribution of the three primary child healthcare systems but not the geographical, political or financial categories. Stand alone outpatient polyclinics provided both general and specialist examinations and treatments in 7 out of 13 former Soviet Union countries and were involved in the management of UTI in children in 15/48 (31%) of all countries. Hospital outpatient services were reported from 4% of countries. Six percent of countries reported that patients suspected of UTI were first seen by nurse practitioners who then referred children with complicated UTI's to a general practitioner, pediatrician or pediatric nephrologist.

**Table 1:** Number and percentage of 48 European countries offering different first contact healthcare services for children suspected of urinary tract infections during regular working hours, at night and on weekends.

Service providers *N* (%)	Weekdays		Weekends
	Day	Night	Day + Night
1. General practitioner	23 (48)	3 (7)	3 (7)
2. General pediatrician	37 (75)	4 (8)	14 (27)
3. Nurse practitioner	3 (6)	2 (4)	1 (2)
4. Polyclinic	15 (31)	18 (35)	21 (42)
5. Emergency room	0	23 (48)	23 (48)
6. Emergency hospital	2 (4)	1 (2)	0
7. Emergency doctor	0	3 (6)	2 (4)
8. Mobile ambulance car	0	1 (2)	2 (4)
9. Pediatric nephrologist	1 (2)	0	0
10. Telephone service	0	1 (2)	0

(1) General practitioner, (2) primary care pediatrician, (3) nurse practitioner, (4) polyclinic (stand-alone), (5) emergency room in general or children's hospital, (6) emergency hospital, (7) emergency doctor with own car, (8) mobile ambulance car (state owned), (9) pediatric nephrologist, (10) telephone service.

During nighttime hours, children with suspected UTIs were evaluated at ten different service levels or pathways (Table 1): 29% of countries provided UTI care in hospital-based outpatient clinics, 11% in freestanding outpatient polyclinics or a combination of both (13%). Primary care pediatricians and general practitioners offered evening and early night (7.00 pm–10.00 pm) services in 27% of countries, however, they were rarely engaged late at night and during weekends. Thus, unlike daytime services nighttime care for suspected UTI did not follow the pattern of the three European primary child healthcare systems. National income, political systems, population size or geographic region did not appear to affect the observed pattern of care during regular office hours, or during nighttime and weekends.

### 3.2 Pediatric rare disease nephrology: pathways of care for children with a first episode of steroid sensitive nephrotic syndrome

Patient pathways at the interface between inpatient and outpatient services were analyzed using a first episode of SSNS as an example. In 33 (69%) of countries, patients who went into remission after a first episode of NS were seen by a pediatric nephrologist in the renal outpatient clinic of the same hospital within three months of hospital discharge. In seven (15%) countries, patients were evaluated either in a hospital outpatient clinic or in external pediatric practices. Countries also reported referrals to external practices or hospital outpatient clinics by regional pediatric nephrologists (*N* = 5), general pediatricians (*N* = 1), adult nephrologists (*N* = 1), or follow-up with patients at a home visit by a nurse from the hospital (*N* = 1).

### 3.3 Pediatric emergency nephrology: pathways of care for children with community acquired acute kidney injury

The treatment pathways for children suspected of AKI in the primary care setting were examined at the interface between primary care and highly specialized hospital kidney care. Pediatric dialysis facilities for the treatment of AKI were available in 47 (98%) of the countries; one country needed cross border care.

During regular office hours, children with AKI were transferred from primary care to secondary care hospitals for further treatment in 51% of the participating countries. Transfers from secondary care hospitals to university pediatric hospitals and other specialized facilities were reported in 36% of countries. In 15% of countries, AKI was treated exclusively in secondary care hospitals unless dialysis was indicated.

University Children's Hospitals were directly or indirectly involved in the treatment of AKI in 58% of reporting countries; 18% of countries reported immediate transfer to tertiary-care children's hospitals, whereas 29% of countries reported collaboration with specialized pediatric nephrology centers and other institutions, including adult nephrology (Table 2).

**Table 2:** Number of countries offering secondary and tertiary healthcare services for children with acute kidney injury in various health areas.

	Day	Night	Weekend
Secondary care hospitals	7	6	6
University hospitals	8	6	6
Pediatric nephrology centers	10	9	9
Combination of all the above	22	22	21
Other^a^	1	5	6
Total	48	48	48

^a^ Includes adult hospitals, polyclinics and emergency hospitals.

Highly specialized pediatric nephrology centers were involved in AKI care in 68% of the participating countries. Twenty-two percent of countries reported immediate referral of AKI patients to specialized pediatric nephrology centers, and 46% reported collaboration with secondary and tertiary care hospitals (Table 2).

At night and on weekends, 44% of countries reported direct referral to specialized tertiary care hospitals that provide around-the-clock service by pediatric nephrologists and acute dialysis facilities in intensive care units. Ten percent of countries reported that AKI was also treated in adult nephrology units and/or emergency hospitals. The need for cross-border care of AKI by neighboring countries was reported from one country. National patterns of care systems showed no significant differences in AKI care according to population size, primary care systems, and political systems.

The main national-level concerns raised in response to an open-ended question about national needs and desires regarding AKI care was that 35% of participating countries called for greater collaboration between pediatric nephrologists and ICU pediatricians (intensivists) (Tables 3A–C). When patients needed to be admitted to intensive care units, only 27% of countries reported that acute dialysis procedures such as continuous renal replacement therapy (CRRT) were performed by closely collaborating pediatric nephrologists, intensive care pediatricians and dialysis nurses. In 23% of countries, intensive care pediatricians collaborated with either dialysis nurses (6%) or pediatric nephrologists (19%). In 25% of countries, intensive care pediatricians managed young kidney patients with the assistance of adult nephrologists. When AKI patients admitted to the ICU required long-term hemodialysis or peritoneal dialysis, pediatric nephrologists were always involved. Ninety percent of countries reported close collaboration in the ICU between pediatric nephrologists, cardiologists, urologists, surgeons, and neonatologists.

**Table 3:** (**A**) Reported top needs for adequate provision of care for children with acute kidney injury in 7 **low-income countries**.

Bosnia	More training of nurses and doctors in dialysis techniques	Summary of problems reported from 7 countries with low income economies. Lack of 1. Specialized medical & paramedical training in dialysis 2. Plasma exchange facilities 3. Acute hemodialysis for young infants & neonates 4. Multidisciplinary team approach in dealing with AKI during an ICU admission (the intensivist has primary role without involving the nephrologist) 5. AKI protocols, framework & biomarkers
Bulgaria	1. Availability of plasma exchange 2. Availability of tests for new markers of AKI	
North Macedonia	1. Organization and referrals 2. Acute hemodialysis for infants and small children	
Romania	1. Multidisciplinary team of intensive care doctors + pediatric nephrologists (at this moment the acute kidney injury patients are treated only by intensive care doctors) 2. Easier access to acute renal therapies (they are accessible only in 5–7 centers across the country) 3. Access to better diagnostic tools for AKI biomarkers	
Turkey	1. Better definition of AKI 2. AKI prevention guidelines in intensive care units 3. Collaboration with intensive care doctors 4. Widespread use of continuous dialysis methods	
Ukraine	1. Treatment of edema during AKI 2. Adequacy of dialysis techniques according to different patients with AKI	
Uzbekistan	1. HUS 2. AKI in hypovolemic shock 3. AKI in acute nephritis and AKI in nephrotic syndrome	

AKI, acute kidney injury; etc.

**Table 3:** (**B**) Reported top needs for adequate provision of care of children with acute kidney injury in 9 **middle income countries**.

Armenia	More ICUs being able to provide RRT	Summary of problems reported from 9 countries with middle income economies. Lack of 1. ICUs offering acute dialysis 2. MDT approach in dealing with AKI during an ICU admission and lack of nephrologists’ involvement) 3. Efficient preventive protocols (e.g., early alert algorithms) & tools assuring optimal pre-emptive management 4. Neonatal AKI: diagnosis, treatment 5. Diagnosis & management of advanced AKI (stage 3) in the primary setting 6. Acute dialysis machines & supplies both for neonates and older children 7. Dialysis (medical & paramedical) training 8. Inadequate financial support for dialysis 9. Inefficient referral process for AKI 10. Adult nephrologists are dealing with pediatric dialysis
Estonia	Better collaboration with PICU	
Greece	1. Appropriate preventive measures 2. Early diagnosis and management 3. More involvement of pediatric nephrologists in dialysis	
Hungary	1. Diagnostics of neonatal acute kidney injury 2. Treatment of neonatal acute kidney injury (acute renal replacement therapy included) 3. Diagnostic and treatment of AKI grade 3 in primary care and the children's hospitals that are not nephrological centers	
Montenegro	1. Education of staff members and better management of patients with AKI	
Poland	1. Improvement of support in good quality medical equipment for acute dialysis in neonates and infants (more suitable/better operating) for both peritoneal dialysis and CRRT (better availability of modern equipment) 2. Early detection of AKI—education of medical staff of other specialties, more available test for early AKI required; Introduction of quick diagnostic AKI tests e.g., nephrocheck test kit 3. Inadequate financial support (reimbursement) for AKI diagnosis and treatment 4. Introduction of systemic tools for prevention of AKI, e.g., NINJA approach to reduce nephrotoxicity	
Portugal	1. Adequate patient referral for recognized reference centers 2. Adequate hemodialysis machines/monitors for younger children (<10 kg)	
Slovakia	Main decision and competence should be on the site of pediatric nephrologists vs. intensivists and adult nephrologists	
Slovenia	Acute hemodialysis modalities should be done by pediatric nephrologist and not adults as currently	

**Table 3:** (**C**) Reported top needs for adequate provision of children with acute kidney injury in **8 high income countries**.

Belgium	Concentrating care in a few highly specialized units	Summary of problems reported from 9 countries with high income economies. Lack of 1. Pediatric workforce 2. Centralized care in reference centers 3. Multidisciplinary team approach in dealing with AKI during an ICU admission (lack of nephrologists’ involvement) 4. NICUs with acute dialysis facilities 5. Electronic observation charts - Communication gaps between primary & secondary care - Shortage of available PICUs - Long waiting time for transfer to a university children's hospital e.g., for HUS patients
Finland	Better identification and treatment of AKI of certain groups of in-hospital patients (neonatology, oncology etc.)	
France	24/7 access to pediatric kidney diseases facilities (more pediatric workforces)	
Germany	Filling the gaps between primary and tertiary care Promoting electronic health records for patients Providing telemedicine services between primary, secondary and tertiary care	
Ireland	Management of hemolytic uremic syndrome	
Sweden	1. Shortage of available pediatric intensive care units 2. Further technical development of neonatal hemodialysis	
Switzerland	1. More rapid transfer from general tertiary hospitals to the university centre in cases of a HUS 2. Earlier involvement of the nephrologist in the PICU	
United Kingdom	1. Better interface/communication between primary and secondary care 2. Neonatal hemodialysis pending research outcomes 3. Electronic observation charts	

One-third of countries reported their needs to accelerate the pathways for diagnosis of AKI in situations such as hemolytic uremic syndromes and acute nephritic syndromes through screening with new biomarkers and application of diagnostic AKI guidelines. In 23% of countries, pediatric nephrologists offered a consultation service of highly specialized nephrology centers to primary and secondary care pediatricians by telephone, including video contact and integrated electronic medical records and telemedicine software (Tables 3A–C).

### 3.4 Responses to all open-ended questions

Pediatric nephrologists from 42 (87%) of 48 European countries indicated that the organization and design of their national pediatric care system would benefit from international discussion and external support under the umbrella of ESPN. The need to establish national patient registries for severe childhood kidney disease was reported through open ended questions. The top three discussion points were (1) improving pediatric workforce planning (60% of the 48 countries), (2) better organization of outpatient kidney care (50%), and (3) improvement of hospital care (58%) for children with acute and chronic kidney disease. The following six countries did not request international support: Finland, Norway, Portugal, Cyprus, Lithuania, and Georgia. Sweden, Germany, and Luxembourg only wanted to discuss workforce planning and staff scheduling.

One-third of the study participants cited the need to improve communication between general pediatricians and pediatric nephrologists and between inpatient and outpatient institutions, and to address gaps in communication between primary and tertiary care. A quarter of representatives from the European countries reported one or more of the following three challenges: (i) a knowledge gap in pediatric nephrology among primary care pediatricians or general practitioners, (ii) lack of collaboration between general practitioners and specialists facilitating timely referrals to specialized kidney centers, and (iii) the overuse of outpatient services out of regular working hours at larger nephrology centers by children with mild kidney disease.

## 4 Discussion

This survey revealed a great variability of patient pathways for European children and young people with UTI, SSNS and AKI during standard working hours, night-time and weekends. Pediatric nephrologists from 48 European countries concluded that the organization and conceptualization of their national pediatric kidney care system would benefit from international discussion under the umbrella of ESPN. The main points for discussion were pediatric workforce planning, designing of outpatient renal care and organizing hospital care for children with acute and long-term kidney disease.

The results of this study are in concert with two previously published ESPN surveys (1, 2) which have shown further diversities of pediatric kidney care resulting in a lack of adequacy, availability accessibility and affordability of healthcare. Identifying diversities in healthcare services helps filling the gaps in order to fulfill the Guidelines of the Committee of Ministers of the Council of Europe on child-friendly healthcare concerning protection, prevention, provision and participation (7). Thus, healthcare services for children with kidney disease need to be reorganized by European healthcare decision makers. Endorsed by the previous publications on diversity, availability of pediatric dialysis facilities for AKI had increased from 83% in 1998 (35 countries included) to 95% in 2017 (44 countries included) and to 98% in 2020 (48 European countries included) (1, 2). Indeed, comparison of general standard pediatric procedures in European countries also revealed national specificities, but there are no simple explanations for their roots, causes and variability (1–4). It turned out that the major differences in child healthcare service systems (CHCSS) were not so much based on scientific facts but on national culture and local traditions (3). When comparing CHCSS in different countries, we showed in one of our previous studies that the presidents of different national pediatric societies—when looking at CHCSS in other countries—assume that any deviation from what they consider to be their medical norm is only because other countries lack the financial resources, knowledge, organization, or will to follow suit (3). This view assumes that everyone in Europe is working toward the same pediatric goals, with some countries being more successful than others (3, 8). However, assuming unlimited financial resources for all European countries, Lyn Payer concluded that national goals in medicine may still not be the same because of differences in national priorities (8).

In an increasingly specialized pediatric care system such as nephrology, the challenge is to design appropriate primary care for children with acute, relapsing or other forms of chronic kidney diseases. Any delay in accurate diagnosis and proper therapeutic intervention exposes young patients with kidney disease to unnecessary disease burden and less favorable long-term outcome.

Immediate diagnosis of bacterial UTI should be relatively easy in most European countries if urine microscopy, urine dipsticks, and culture media were available in all facilities. Standardized empiric antibiotic therapy based on regional antibiotic resistance rates of the most common pathogens would greatly improve treatment success and outcome. In fact, proper initial care can be provided by all pediatricians and family physicians while hospital admission will still be needed for the sickest patients, particularly the youngest ones, in whom underlying anatomical urinary tract problems may be present. In contrast to adult nephrologists, only a small minority of fully trained European pediatric nephrologists provide primary care in independent practices (1). One could argue that an unknown proportion of parents also bring their adolescent children to the practices of urologists or adult nephrologists in the cities, or to Feldschers and nurse practitioners in rural areas. Unfortunately, we were neither able to provide statistics on their role in the primary care of children with kidney disease as part of this study nor did we analyze the difference of care pathways between urban and rural areas. The question is whether the complexity of more than three alternative treatment pathways for children with UTI offers an advantage compared to a system with only two or three types of providers based on local needs and preferences. An article from the European Academy of Pediatrics on treatment modalities for UTI concluded that “simple, brief, practical, and easy-to-remember guidelines and educational strategies should be developed to ensure their implementation” (9). Our survey found that the number of care pathways was smaller outside regular working hours. However, this did not mean that renal services had become more appropriate, as pediatricians and general practitioners were far less involved at night and on weekends. Thus, the chances of young patients being seen by experienced physicians outside of regular working hours were less. In addition, secondary and tertiary care hospitals suffered from extra work because their emergency departments were overcrowded with children with mild kidney problems. We conclude that national guidelines should provide guidance to all physicians on the optimal management of UTIs.

Our results show that in most countries, outpatient care of children with SSNS was professionally provided by pediatric nephrologists working in children's hospitals. Thus, they can be considered gatekeepers for children with frequent relapses who may need additional psychosocial care. The challenge in an increasingly specialized hospital system is to design pediatric hospitals that are appropriate for long-term ill children with complex and complicated needs (10), whereas the “simplest” cases can be treated outside the tertiary setting.

Multidisciplinary treatment of children with AKI affected by damage of extra renal organs and tissues demonstrates how procedures such as comprehensive pediatric assessment can improve treatment and outcomes. Selewski et al. (11) concluded that “coordinated multidisciplinary efforts are urgently needed to raise awareness of pediatric AKI and educate pediatric primary and subspecialty care.” Kaddouragh et al. (12) wrote, “Recent epidemiologic data clearly show that a large proportion of hospitalized children have AKI on admission, suggesting that AKI often begins in the community.” In Europe in the 1990s, the annual incidence of AKI was 8 per 1 million total populations (13). Epidemiologic data suggest that the incidence of AKI is increasing worldwide and that AKI is more likely to occur in children in low-income countries (14). In 2019, approximately 1,800 pediatric nephrologists and an unknown number of pediatric dialysis nurses were performing all types of acute and chronic dialysis in European children (2). In fact, there are also pediatric intensive care physicians performing acute dialysis in children in pediatric intensive care units, however, to our knowledge their actual number was unknown in 2020. Data reported in our survey by members of ESPN confirm the need for earlier intervention and optimal use of acute dialysis in children with worsening renal function during AKI episodes (14, 15). The most common problem seems to be the lack of highly specialized pediatric workforce in many countries, irrespective of low or high income countries. Areas of requests were dealing with networking with other subspecialties, new technologies, digital pediatrics and artificial intelligence.

Our study has a few limitations. One is the possibility of subjectivism when answering some of our questions such as the listed top three priorities. Second, we had chosen only one single reporting person from each country, the President of the National Pediatric Nephrology Association or a senior pediatric nephrologist. When it comes to smaller countries, the answers are very credible, while the situation with larger countries may be more complicated because of regional diversity within a given country. We have always insisted that when there were doubts about generalizing answers, the reporting nephrologists should consult with their pediatric nephrologist colleagues or pediatric society.

Given that the European political scene is very inhomogeneous, it is possible that certain weaknesses of the health system and the provision of renal care for children were not reported by the reporters in order not to offend his/her health authorities. Theoretically, we could have avoided subjectivism in the answers and a critical approach to the questions if our survey had been anonymous. However, this would have entailed further disadvantages for the credibility and accuracy of the data. The great advantage of our survey is the close cooperation and mutual trust of all co-authors and their civil courage to present reliable data.

The value of our study consists in the fact that the results can be compared with previous studies from the same region, in this case with another ESPN study as well as with other international studies. Given that the interval between the second and third ESPN survey was very short, we did not present recent statistical data of workforce and medial supply chains. However, we are planning to publish the results from the remaining 8 out of 15 questions of our survey in a separate article. In order to compare our European results with the results of surveys on these issues in non-European countries, we did extensive web research to see if there are similar surveys and results. We used the keywords: “urinary tract infection” (UTI), “steroid sensitive nephrotic syndrome” (SSNS), “acute kidney injury” (AKI), “pathways “and “referrals” in various combinations but we have not find any work that addresses these entities in relation to referral pathways and services that provide renal care to pediatric patients, during regular working hours, night shifts and weekends. The articles we found were mostly related to clinical pathways such as diagnostic methods which was not the subject of our work (16). According to our best knowledge, our study is the only one that deals with referral pathways for pediatric kidney care and this is one of the strengths of our work.

## 5 Conclusions

Clinical pathways (CPW) in primary care and hospitals are tools for guiding evidence-based health care that need to be proposed internationally and adapted locally to national desires and needs. Clinical pathway guidelines are a major challenge because there are few national studies on the quality of patient pathways and their impact on health care quality. The European Pathways Association has described the characteristics of clinical pathways (17), but to our knowledge there is still no standardized guideline on what actually constitutes CPWs in pediatric nephrology. As the findings from this survey make clear, the focus should now be on comparing experiences internationally to enable the planning, redesign, and implementation of new strategies in Europe. We conclude that health systems are widely unable to respond to and fill gaps in care for children with kidney disease outside of normal working hours. It is conceivable that these difficulties are not limited to pediatric nephrology but also apply to other pediatric subspecialties. In other words, key areas of health care are challenged to make fundamental decisions about the use of innovations and to rethink their implementation.

Many threats to health and health systems—whether economic, environmental, social, or epidemiologic—require European collaboration (18, 19) and complex systems thinking (6) The public good and benefits arising from such cooperation may require a certain pooling of resources. All European pediatricians could strive to learn from this and previous ESPN surveys (1, 2) and strengthen national health systems to better withstand old and new crises.

## Data Availability

The datasets presented in this article are not readily available because not applicable. Requests to access the datasets should be directed to Jochen Ehrich.

## Glossary

CHCSS, child health care service system; CPW, clinical pathways; PCPeds, primary care pediatricians; GPs, general practitioners; PPC, primary pediatric system with more than 75% of children <18years under the care of pediatricians; GPC, general practitioner/family doctor system with >75% of children seen by GPs; MiC, mixed care system with pediatricians and general practitioners providing primary care to 25%–75% of children; ESPN, European Society for Pediatric Nephrology; UTI, urinary tract infection; SSNS, steroid sensitive nephrotic syndrome; AKI, acute kidney injury; CKD, chronic kidney disease.
