# Supplementary material for: Diversity of kidney care referral pathways in national child health systems of 48 European countries
Source: Front Pediatr. 2024 Jan 16;12:1327422. doi: 10.3389/fped.2024.1327422 (PMC10825019; doi:10.3389/fped.2024.1327422)
Supplement: Supplementary file 1 [file Datasheet1.pdf]

Questionnaire on European paediatric renal care in 2020

1. How is first access care for children with a bacterial urinary tract infection (UTI) organised in your country? *Please click and explain (if necessary) the patient's pathways shown below on "who, where, when, how, why" is providing the care of UTI?"*

During day-time and working hours, the patient is contacting most frequently first:

- a general practitioner Yes/No
- a primary care paediatrician Yes/No
- a nurse practitioner Yes/No
- a policlinic Yes/No
- if also other, please specify in a few words the care giver or any other relevant information:

During night-time and outside working hours, the patient is contacting most frequently first:

- a general practitioner Yes/No
- a primary care paediatrician Yes/No
- a nurse practitioner Yes/No
- a policlinic Yes/No
- if also other, please specify the care giver

During weekends the patient is contacting most frequently first:

- a general practitioner Yes/No
- a primary care paediatrician Yes/No
- a nurse practitioner Yes/No
- a policlinic Yes/No
- if also other, please specify the care giver

2. What is the pathway of a 4-year-old patient with a first episode of a steroid sensitive nephrotic syndrome after leaving hospital? Who will provide the first outpatient check up examination during the next 3 months? *You may click more than one answer.*

1. The out-patient clinic of the same children's hospital? Yes/No
2. A primary care paediatrician? Yes/No
3. An adult nephrologist in private practice? Yes/No
4. if also other, please specify the care giver

3. If acute kidney injury (AKI) is suspected during consultation in a primary care setting, how is emergency care for children with AKI organised in your country?

During day-time and working hours, the patient is contacting most frequently first:

- a secondary care children's hospital
- a university children's hospital
- a highly specialised paediatric nephrology centre

- an adult nephrology unit
- if also other, please specify the care giver

During night-time and outside working hours, the patient is contacting most frequently first:

- a secondary care children’s hospital
- a university children’s hospital
- a highly specialised paediatric nephrology centre
- an adult nephrology unit
- if also other, please specify the care giver

During weekends the patient is contacting most frequently first:

- a secondary care children’s hospital
- a university children’s hospital
- a highly specialised paediatric nephrology centre
- an adult nephrology unit
- if also other, please specify the care giver

Please explain the patient’s pathways if you have come to the conclusion that the proposed multiple-choice questions will not correctly reflect the strengths and weaknesses of your services (who, where, when, how, why is providing the care of AKI?):

4. Please list the top three priorities of acute kidney disease entities requiring urgent changes of current treatment strategies in your country.

5. Please list the top three <u>un</u>solved problems of child health renal care that have been identified in your national health care system (e.g. availability and accessibility of paediatric nephrology centres).

<u>6.</u> What does your national society expect from ESPN in the near future?

- More training courses
- More webinars
- More congresses
- if other, please specify your wishes

7. What should become the next top three priorities of ESPN for improving paediatric nephrology in the whole of Europe?
